# Impact of cognitive behavioral therapy on premature ejaculation patients: A prospective, randomized controlled trial protocol

**DOI:** 10.1371/journal.pone.0295663

**Published:** 2023-12-27

**Authors:** Qiyun Yang, Hongcai Cai, Zi Wan, Min Chen, Bicheng Yang, Yun Xie, Yadong Zhang, Xiangzhou Sun, Jia Tang, Ming Kuang, Hongying Liu, Chunhua Deng

**Affiliations:** 1 The First Affiliated Hospital, Sun Yat-sen University, Guangzhou, China; 2 Hangzhou Kang Ming Information Technology Co., Ltd, Hangzhou, China; University Hospital of Münster, GERMANY

## Abstract

**Background:**

Premature ejaculation (PE) is one of the most common male sexual dysfunctions, with a prevalence of about 4%-39% in the Chinese population. Studies have shown that a variety of biological factors can lead to premature ejaculation, such as central nervous system disorders, hypersensitivity of the penis head, and psychological factors. Based on clinical experience, psychological counseling and education of patients and partners should be ranked as the first priority when treating PE. Cognitive behavioral therapy (CBT) addresses emotional, behavioral, and cognitive disorders by altering beliefs and actions. It has also been demonstrated to be clinically useful in treating a number of diseases. The purpose of this trial is to evaluate the efficacy of a mobile-based CBT intervention on patients with PE compared to conventional routine treatment.

**Methods:**

This study is a prospective randomized controlled trial that will be conducted from May 2023 to Dec 2024 at ten hospitals, primarily including the First Affiliated Hospital of Sun Yat-sen University with an 8-week follow-up. The clinical trial central randomization system will be used to create and implement the specific randomization method. Baseline data of both groups will be measured and collected. The premature ejaculation diagnostic tool (PEDT) and the female sexual distress scale-revised for premature ejaculation (FSDS-R-PE) will be collected on the first day, 28±2 days, and 56±2 days during the intervention period, and the intravaginal ejaculatory latency time (IELT) will be measured in both groups. The Shapiro-Wilk test will be used for normality testing. Pearson correlation analysis will be used for correlation analysis. Differences between groups will be compared using analysis of variance or exact probability calculations.

**Discussion:**

This study will investigate the effect of a mobile-based CBT intervention on patients with PE.

**Trial registration:**

Chinese Clinical Trial Registry (ChiCTR2300070581).

## Introduction

Premature ejaculation (PE) is the most common male sexual dysfunction, which refers to early ejaculation during sexual intercourse. PE is generally classified into primary PE (LPE), secondary PE (APE), natural variable PE (NPE), and subjective PE (SPE) [1]. In Chinese population, the prevalence of PE ranges from approximately 4% to 39% [2–4]. Approximately 26.0% of PE patients report experiencing PE, with LPE accounting for 12.3%, APE accounting for 18.8%, NPE accounting for 41.1%, and SPE accounting for 24.8% [4]. Currently, the diagnostic criteria for PE mainly consider factors such as ejaculation time, control, and sexual satisfaction. The etiology of PE may involve both physiological and psychological factors. Early evidence suggests that PE has a psychological or interpersonal basis, largely due to regulatory changes resulting from anxiety or hasty sexual experiences. Compared to healthy controls, PE has a negative impact on patients’ confidence, self-esteem, and sexual relationships and may cause anxiety [5], embarrassment, and even depression [3]. Compared to the other three types of PE, the psychological factors in APE are more prominent, and patients require etiology-specific treatment such as psychotherapy or pharmacotherapy [1]. Research has shown that after psychological intervention, PE patients had statistically significant improvements in post-ejaculatory reflex control, sexual satisfaction for both the patient and their partner, and scores for sexual activity anxiety or depression compared to the control group [6]. Therefore, this study will focus on APE patients and aim to identify an effective psychological intervention that can improve their psychological health and quality of life.

In clinical practice, the diagnosis and evaluation of PE patients mainly include the collection of medical history, physical examination, auxiliary examination, assessment of scales, and intravaginal ejaculatory latency time (IELT) evaluation. Drug therapy, behavioral therapy, and sex therapy are the main methods of PE treatment, but the effectiveness of a single therapy is poor. The latest guideline recommended combining drug therapy and cognitive-behavioral therapy (CBT) in a couple-centered approach as a new combination therapy [7]. Unlike psychotherapy, CBT is a problem-centered and action-oriented therapy used to manage out-of-control emotions, behaviors, and cognition. The clinical efficacy of CBT has been demonstrated and used to treat diseases in clinical and non-clinical settings, such as personality conditions, psychological disorders, and behavioral problems. The latest randomized controlled study showed that after 6 weeks of CBT intervention, the sexual distress and sexual satisfaction of couples with sexual dysfunction were significantly improved compared to the control group. Although the sexual relationship satisfaction decreased after 6 months of intervention, the improvement of sexual distress in patients was sustained [8]. In addition, cognitive-behavioral sex therapy (CBST) has been widely applied in the treatment of erectile dysfunction (ED) in men. The adjunctive use of CBT has been shown to have long-term beneficial effects on ED patients receiving Phosphodiesterase Type 5 Inhibitor (PDE5i) treatment [9]. Another study showed that the CBST group had significantly improved International Index of Erectile Function-5 (IIEF-5) scores and reduced depression scores compared to the placebo group after treatment [10].

However, in the field of sexual dysfunction treatment, especially in the treatment of premature ejaculation (PE), many patients may be reluctant to seek treatment at hospitals or clinics due to shame or self-esteem issues. Therefore, mobile-based treatment has become a new option. In recent years, several clinical studies on remote disease management based on WeChat technology have been conducted in China, and the results have shown that the patient disease remote management model, which is based on in-hospital treatment and aims to strengthen out-of-hospital management, has improved patient treatment effects and clinical outcomes to some extent compared with traditional routine treatment models [11–13]. Against this backdrop, we integrated various intervention methods using the CBT approach and aimed to use data technology to intervene with patients and promote cognitive and behavioral changes in them. Ultimately, we designed a mobile medical application based on WeChat Mini Program to be used in conjunction with drug or equipment therapy. The purpose of this study is to evaluate the clinical efficacy of mobile-based CBT in the treatment of PE. Meanwhile, we will explore suitable means and application scenarios for remote management during the research process, and hope to provide reference for the development of guidelines for remote management of premature ejaculation.

### Evidence before this study

We conducted a search on PubMed for studies using CBT in patients with PE from November 2016 to April 2023. The search terms included PE, sexual dysfunction, and CBT, and Medical Subject Headings (MeSH) terms were used to include synonyms. Based on our search, some studies have shown the benefits of using a combination of psychological and medical therapies to treat PE, but systematic meta-analysis data indicate inconsistent effects of psychological/behavioral interventions on treating PE. CBT has been shown to be effective for performance anxiety/social anxiety (PA/SA) and is recommended for sexual performance anxiety (SPA), but lacks controlled studies [14]. Furthermore, we did not find objective evaluation results of CBT in the PE population. We identified two randomized controlled studies on the efficacy of CBT for patients with sexual dysfunction. The results showed that CBT was an effective and supportive adjunctive treatment for Pakistani men with erectile dysfunction (ED) who were taking type 5 phosphodiesterase inhibitors, and the treatment effect lasted longer [9]. Another randomized controlled study investigated the effect of CBT on the sexual function, marital adjustment, depression, and anxiety levels of women with vaginal spasm and their husbands. The results indicated that CBT was an appropriate treatment for vaginal spasm and had beneficial effects on the sexual function, marital adjustment, depression, and anxiety levels of both women and their husbands [15].

### Added‑value of this study

Currently, sexual psychological counseling is generally recommended as the primary treatment for PE. Previous research has mainly focused on patients with ED, and limited information is available regarding the impact of CBT on the PE population. Although recent reports have suggested combining drug therapy with sex-focused CBT when possible for treatment [16], this form of therapy has yet to be validated by evidence-based medicine. Our study aims to evaluate the specific therapeutic effects of CBT in the treatment of PE through clinical research and provide support for the advancement of subsequent psychological treatment methods.

### Objectives

Aim 1: To determine the efficacy of mobile-based CBT intervention compared to conventional treatment for patients with premature ejaculation (PE).

• Hypothesis 1a: a significant difference in the mean scores of the premature ejaculation diagnostic tool (PEDT) is expected between the two groups within 8 weeks.

### Trial design

This study is a multicenter, randomized, controlled, open-label parallel-group exploratory trial, consisting of an intervention group and a standard treatment group. Participants will be randomly assigned in a 1:1 ratio to evaluate the efficacy of CBT in improving ejaculatory control in patients with PE. The study protocol follows the Standard Protocol Items: Recommendations for Interventional Trials (SPIRIT) [17].

## Method

### Study setting

The study will be conducted from May 2023 to Dec 2024 at ten hospitals in China, primarily including the First Affiliated Hospital of Sun Yat-Sen University.

### Eligibility criteria

Patients meeting the following inclusion criteria will be recruited: (1) male aged between 18–40 years; (2) in a stable heterosexual relationship with the same partner for at least 6 months; (3) diagnosed with PE according to the ISSM definition and not medically managed for at least 7 days; (4) able to use mobile devices such as smartphones and tablets; and (5) having read and agreed to sign the informed consent form.

Patients’ exclusion criteria are as follows: (1) primary premature ejaculation patients; (2) patients taking medications such as amphetamines, dopamine, etc. that may cause PE; (3) patients with cognitive impairment, communication disorders, visual impairment, hearing impairment; (4) patients with other complex male genital diseases; (5) patients with malignant tumors; (6) patients participating in other clinical trials; (7) patients deemed unsuitable for this study by the researchers.

The researchers will conduct preliminary eligibility screening and collect written informed consent from selected participants prior to baseline evaluation. If a data-collection statement is not included in the initial informed consent form for the primary clinical trial, each participant in the ancillary study has to sign a separate consent form. This trial does not involve collecting biological specimens for storage.

### Interventions

**Fig 1:**
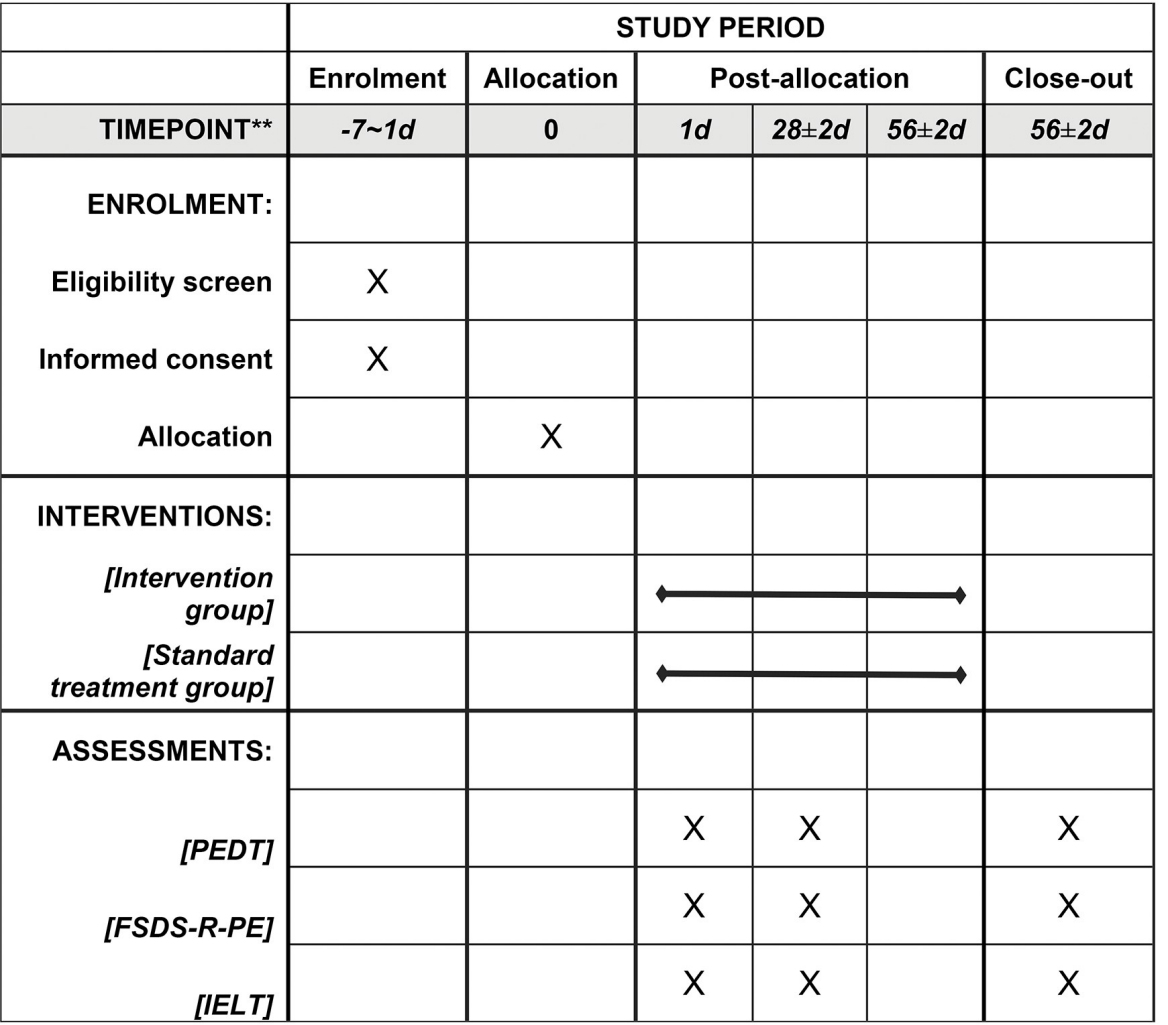
SPIRIT schedule. The intervention group will receive WeChat management and standard treatment. Evaluation of -7-1d will be performed prior to treatment intervention.

**Fig 2:**
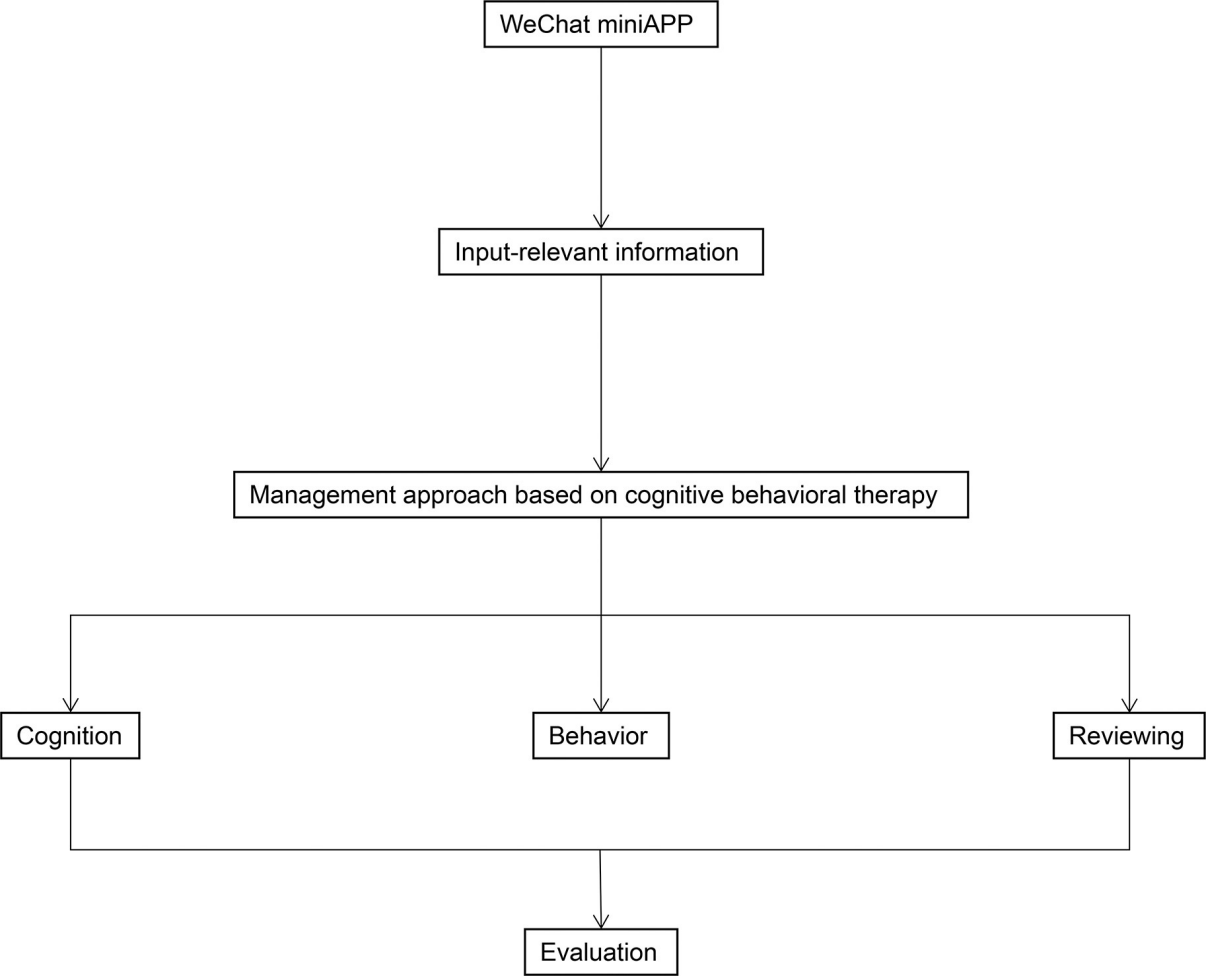
Flowchart of treatment for PE patients.

Patients in both groups will receive conventional treatments, including chemotherapy, traditional Chinese medicine, and instrument therapy, with treatment selection and plans based on the guidelines for diagnosis and treatment of premature ejaculation. To maximize the effectiveness of CBT interventions based on mobile devices in PE patients, we will use remote management through social media software (WeChat) to address sensitive issues that may be difficult to reach in the population. We will use the WeChat-MiniApp: "PE-CBT" developed by Hangzhou Kang Ming Information Technology Co., Ltd. to remotely manage patients of the intervention group. WeChat management mainly includes: (1) based on CBT as the methodology, following the logic of cognitive restructuring, behavioural activation, the goal of exposure and problem solving, the PE-exclusive medical information such as disease knowledge, behavioural training and psychological counselling will be produced into videos and graphics, with a total of 8-week courses arranged; (2) researchers can track patient management through outpatient clinics (Fig 1). As shown in the Fig 2. The entire therapy session is divided into three stages. The stage 1 focuses on improving cognition, the stage 2 focuses on improving behavior, and the stage 3 focuses on reviewing.

#### Criteria for discontinuing or modifying allocated interventions

Participants will be considered for withdrawal from the study under the following circumstances. However, even if participants do not withdraw their consent to participate in the study, they will no longer be considered as trial subjects and will not be included in the analysis of the study as soon as they withdraw from treatment under the following circumstances:

(1) If participants voluntarily withdraw (including dropping out, actively withdrawing, etc.);

(2) If the researcher requires the participant to withdraw due to disease deterioration or other reasons;

(3) If the researcher considers the participant no longer suitable to continue participating in the study. It refers to other conditions not listed in the exclusion criteria, and the investigator believes that the patient is not suitable to participate in the trial due to the comprehensive consideration of the patient’s treatment, life and other aspects.

#### Strategies to improve adherence to interventions

To ensure patients’ compliance with the study, the research team will adopt a grid-based approach, such as telephone and internet reminders, to prompt patients to attend follow-up appointments at the outpatient clinic before the scheduled time. Meanwhile, during each follow-up visit, the research team will emphasize that patients in the intervention group should complete the relevant WeChat learning according to the requirements.

#### Relevant concomitant care permitted or prohibited during the trial

Both groups will receive standard pharmacological treatments.

#### Provisions for post‑trial care

Participants are unlikely to be harmed in the context of patient management based on mobile platforms. In the event that potential harm is identified, the principal investigator will take measures to mitigate the risk. Should any harm occur, the research team will provide appropriate compensation as necessary.

### Outcomes

#### Primary outcome measures

The mean PEDT scores of patients of two groups will be recorded within 8 weeks. To assess treatment effectiveness, patients will be evaluated using a medical questionnaire during each outpatient/telephone follow-up on day 1, at the end of week 4 and week 8 of the study. The questionnaire consists of five questions with five different response categories (A = 0, B = 1, C = 2, D = 3, E = 4) to evaluate the conditions over the past 6 months. Scores <8 mean no problem; scores between 9 and 10 suggest a need for diagnosis at a hospital, while scores >11 indicate premature ejaculation. The PEDT questionnaire has been widely used in China and has demonstrated good internal consistency, reliability, and validity, which has good predictive ability in Chinese premature ejaculation patients [18].

### Secondary outcome measures

(1) PEDT scores of the two groups at 4 and 8 weeks;

(2) IELT duration of the two groups at 4 and 8 weeks;

(3) Female sexual distress scale-revised premature ejaculation version (FSDS-R-PE) scores of the two groups at 4 and 8 weeks.

According to clinical experience, IELT mostly uses self-estimation. To improve the accuracy of self-estimation, the number of penis twitching in the vagina can be used to estimate. At each follow-up visit, participants and their patients will be questioned separately, and each will be asked to provide an independent estimate of IELT.

The FSDS-R-PE is a 13-item questionnaire with 5 response categories for each item (0 = never, 1 = rarely, 2 = occasionally, 3 = frequently, 4 = always), designed to evaluate the sexual function status of female partners of men with premature ejaculation [19]. A higher score indicates a higher level of dissatisfaction with sexual life.

### Participant timeline

**Fig 3:**
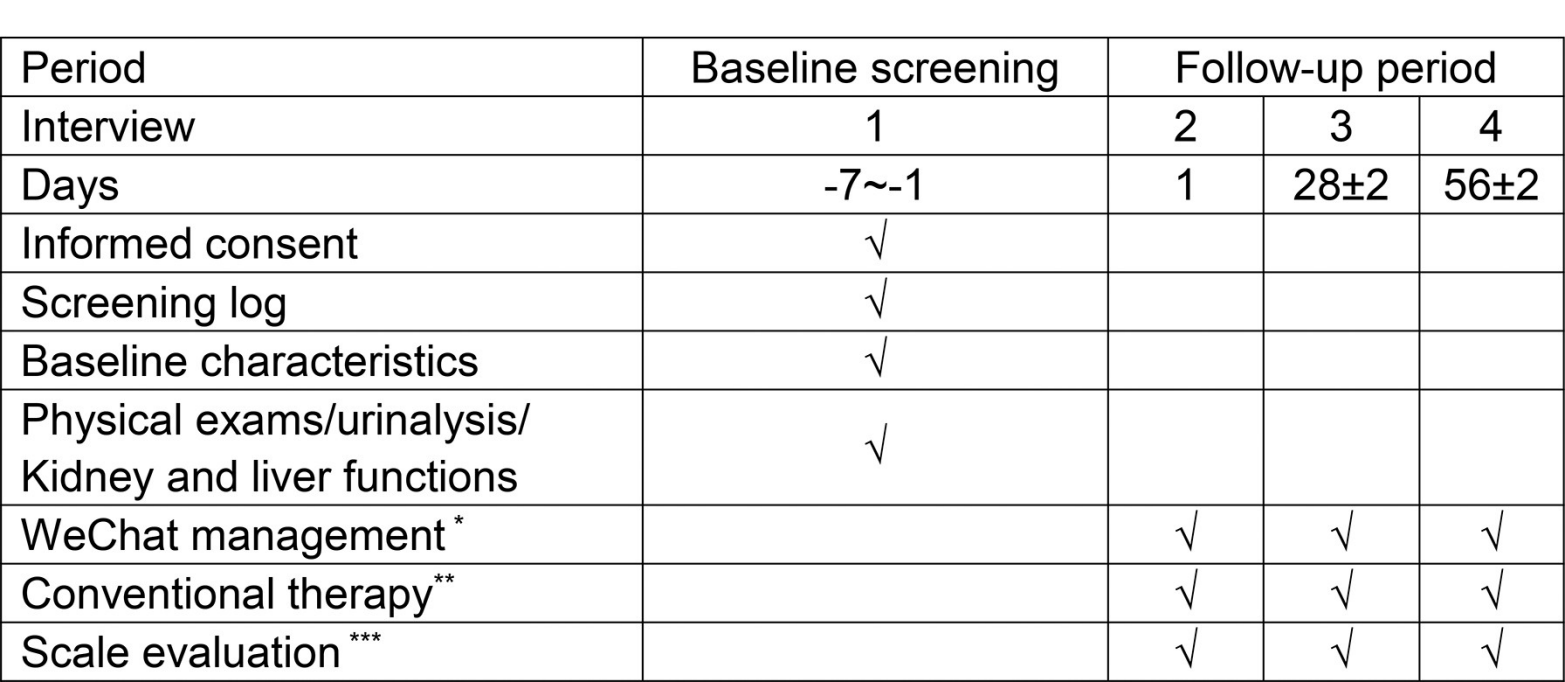
Participant timeline. *Only Group A will accept WeChat management, **Group A/B will receive the conventional therapy, ***PEDT and FSDS-R-PE scales will be completed at each outpatient/telephone follow-up.

The participant timeline is shown in Fig 3.

### Sample size

This study will adopt two groups, the intervention group and the standard treatment group, with a 1:1 ratio for enrollment. The study hypothesizes that the intervention group will have a 30% greater reduction in PEDT than the standard treatment group after 8 weeks of treatment (α = 0.05; β = 0.2). With a maximum follow-up loss rate of 20% for each group, a target sample size of 300 patients, with 150 patients in each group, is determined.

### Recruitment

Participant recruitment has begun in May 2023 and is expected to be completed in Dec 2024. We will recruit participants at ten hospitals in China, primarily including the First Affiliated Hospital of Sun Yat-Sen University. Once recruited, the researchers will provide instructions on how to use WeChat-MiniApp.

### Assignment of interventions: Allocation

#### Sequence generation

In this randomized controlled trial, participants will be randomly allocated to intervention group or standard treatment group at a ratio of 1:1. Allocation will be standardized through the establishment of a randomization system. Researchers will access the system by inputting their account and password. A number generated from patient information inputted will be used to track each patient.

#### Concealment mechanism

Allocation concealment will be ensured through the following mechanism. Designated personnel at the trial center will input basic information of eligible patients into the randomization system according to the randomization table. A random number will then be generated. Patients who meet the inclusion criteria will undergo random allocation after obtaining signed informed consent.

#### Implementation

The allocation sequence will be generated by the centralized randomization system. The investigator will be responsible for patient recruitment. The Trial Management Committee (TMC) will allocate interventions to participants.

### Assignment of interventions: Blinding

#### Who will be blinded

This trial will adopt a non-blinded design, in which participants and researchers will know the interventions assigned to them in this open-label trial. However, not all outcome assessments will be blinded due to the nature of patient-reported outcome measurements. In the analysis process, statisticians will be blinded to the allocation.

#### Procedure for unblinding if needed

The procedure for unblinding is not applicable as this is an open-label trial.

#### Ethics approval and consent to participate

The implementation of this trial protocol will adhere to the ethical principles of the 1975 Helsinki Declaration, and the research design, protocol, information sheet, and informed consent form have been approved by the Ethics Committee of the First Affiliated Hospital of Sun Yat-sen University ([2022]043).

### Data collection and management

#### Plans for assessment and collection of outcomes

Data collection will occur at four time points: baseline, day 1 of intervention period, 28±2 days, and 56±2 days. The collected data will be stored in the form of electronic case report forms (CRFs). Clinical research coordinators (CRC) will complete the electronic CRFs. Researchers will be informed of any changes/edit checks completed by the CRC and the system automatically. Throughout the study process, authorized researchers will be allowed to access the study computer and assess the data.

#### Plans to promote participant retention and complete follow-up

If any missing data is detected via the randomization system, the researcher will contact the participants by telephone.

#### Data management

The research team will be responsible for self-monitoring the study process and data. Such self-monitoring can ensure the smooth running of the trial and identify and mitigate issues before being discovered by monitoring entities. Internal monitoring includes monitoring of appropriate informed consent forms/records, eligibility criteria, data quality, and so on. Monitoring is typically conducted by designated personnel from those involved in the study to identify issues and improve processes. When analyzing the data, a unique identification code will be generated to ensure security. Erroneous or missing data information will be sent to the data manager (DM) in a data query report. Upon receipt of the inquiry, the DM will verify the original records to determine the correction. The original files and signed informed consent forms will be kept in a locked filing cabinet.

#### Confidentiality

Personal information will be collected, shared and maintained in accordance with the ICHGCP.

#### Plans for collection, laboratory evaluation and storage of biological specimens for genetic or molecular analysis in this trial/future use

No genetic or molecular analysis will be conducted in this trial or for future use

### Statistical methods

#### Statistical methods for primary and secondary outcomes

All data will be entered by two members separately and analyzed after confirming that the data is completely consistent. We will use SPSS 22.0 for statistical analysis. For metric data, Shapiro-Wilk test will be used to check for normality. If it meets the normal distribution, it will be described by mean ± standard deviation; otherwise, it will be described by median (P25, P75). Pearson correlation analysis will be used to evaluate the correlation. Metric data of different groups will be described statistically using mean, standard deviation, median, minimum value, and maximum value. Count data of different groups will be described statistically using frequency (composition ratio). We will use exact probability calculation or non-parametric test to analyze the changes before and after intervention in each group. Regarding drop-out analysis, we will describe the actual number of enrolled subjects, excluded subjects, and drop-out subjects one by one, and analyze the specific reasons for drop-out and exclusion. The balance analysis of baseline values will be compared using analysis of variance or exact probability calculation for demographic data and other baseline indicators to measure the balance of each group. Repeated measures information will be expressed as mean ± standard deviation, within-group comparisons will be made using repeated measures analysis of variance, and between-group comparisons will be made using multifactor analysis of variance (MANOVA). We will use P values to determine the efficacy of CBT in PE patients, and P<0.05 will be considered statistically significant. We will list adverse events that occurred during the trial and describe the changes in laboratory test results before and after the trial, and the relationship with the intervention when abnormal changes occurred.

#### Interim analyses

There will be no interim analysis.

#### Methods for additional analyses (e.g. subgroup analyses)

No additional analysis will be conducted.

#### Methods in analysis to handle protocol non-adherence and any statistical methods to handle missing data

In accordance with the ICH-E9 statistical principles for clinical trials, the full analysis set (FAS) will be included all randomized subjects. Depending on the timing of the assessment, each per-protocol (PP) analysis set will consist of patients who complete the 8-week follow-up examination. The FAS will be used as the basis for preliminary analysis. If the difference between the FAS and PP samples exceeds 10%, the PP analysis set will be analyzed again. The safety population includes FAS patients. Unless otherwise stated, all analyses will be performed on both the FAS and PP analysis sets. No replacement or supplementation will be made for missing values. All analyses will be based entirely on observed cases. Patients lost to follow-up will be considered as having dropped out of treatment. Only PP patients will be used for sensitivity analysis to test the impact of this assumption.

#### Plans to give access to the full protocol, participant level-data and statistical code

The results of the clinical trial will be disseminated in the form of a manuscript, although the specific publication venue and timeline have yet to be determined. All relevant data and information will be made publicly available upon publication of the manuscript.

### Oversight and monitoring

#### Composition of the coordinating centre and trial steering committee

*Design and implementation of the study*. Preparation and revision of the protocol; development of the Investigator’s Brochure (IB) and CRFs; publication of the study report; composition of the Trial Management Committee (TMC) members.

*SC (Steering Committee)*. Final protocol agreement; patient recruitment and communication with the Principal Investigators (PIs); review of trial progress and approval of necessary adjustments to the trial protocol and/or IB to facilitate the smooth conduct of the study. The Steering Committee will include all major PIs. One regional coordinator will be selected from the main PIs at each study center.

*Trial Management Committee (TMC) (Principal Investigators*, *study physicians*, *and administrative personnel)*. Study planning; organization of Steering Committee meetings; reporting of adverse events to the National Adverse Event Monitoring Center; responsible for trial master file; budget management and contract negotiations with each center. Randomization; data validation. The TMC will review feedback forms for 12 months and arrange on-site visits.

*Data manager*. Responsible for data entry and maintenance of the trial IT system; data validation.

*Principal Investigators*. Patient recruitment; data collection; completion of CRFs; follow-up of study patients and compliance with the study protocol and IB. Each participating center will be assigned a lead PI responsible for patient identification.

#### Composition of the data monitoring committee, its role and reporting structure

There will be no data monitoring committee for this study because the intervention in this study is considered a low-risk intervention.

#### Adverse event reporting and harms

The type and frequency of any adverse events will be reported to the China National Adverse Reaction Monitoring Center.

#### Frequency and plans for auditing trial conduct

The review process will comply with the International Conference on Harmonisation of Technical Requirements for Registration of Pharmaceuticals for Human Use (ICH) Good Clinical Practice (GCP) guidelines and regulatory requirements. The steering committee will decide on any revisions to the protocol. The revised protocol will also be submitted to the Ethics Committee of the First Affiliated Hospital of Sun Yat-sen University, and reported to the participants when necessary. The results of this study will be disseminated to the public at academic conferences. The authorship will be determined by the steering committee based on each member’s contribution, and the author order will reflect their respective contributions.

#### Plans for communicating important protocol amendments to relevant parties (e.g. trial participants, ethical committees)

The steering committee will decide on any revisions to the protocol. The revised version will also be submitted to the Ethics Committee of the First Affiliated Hospital of Sun Yat-sen University and reported to the participants when necessary.

### Dissemination plans

The results of this study will be presented to the public at academic conferences. The authorship will be determined by the steering group, with the order of authors based on each member’s contributions.

## Discussion

This article provides a detailed description of a parallel randomized controlled trial to evaluate the effectiveness of mobile-based CBT for patients with PE. Currently, the mainstream treatments for PE are psychotherapy, behavioral therapy, and medication therapy. While medication therapy is superior to standalone psychotherapy in reducing PE symptoms, behavioral and psychotherapies offer potential advantages, including minimal side effects and improvement in sexual interaction with partners. To better facilitate sexual psychological interventions, we believe that mobile-based CBT intervention is a feasible approach that can increase patients’ PEDT scores. We believe that mobile-based CBT can effectively improve sexual satisfaction for both male PE patients and their female partners. The significance of this study is that no report on the effectiveness of CBT in PE patients has been available to date. Due to the high accessibility and availability of mobile-based CBT interventions, the intervention is expected to improve the treatment level of PE patients. In addition, this study may demonstrate the effectiveness of CBT-based sexual psychological and behavioral interventions in prolonging IELT in PE patients and serve as a guide for future sexual psychological and behavioral interventions for PE patients. However, this study has certain limitations. This study excludes primary PE patients, patients with abnormal causes of PE, and patients who cannot use mobile phones, and only included some appropriate PE patients. Future studies may involve larger patient populations. Secondly, due to the nature of the study, blinding cannot be fully employed in psychotherapy research, and CBT treatment for premature ejaculation requires doctors’ meticulous observation and treatment of patients. Therefore, WeChat-based remote management may affect treatment effectiveness. Previous studies have shown that standardized non-pharmacological interventions (such as pelvic floor muscle training) can play a positive role in the treatment of PE patients, and the digital intervention in this study is also standardized which is in line with clinical practice. However, due to the particularity of PE disease (patient privacy and high sensitivity), digital intervention may not be suitable for all patients in the intervention group in this study. Based on the deep analysis of these research results, the adaptability problems about the digital intervention will be further discussed.

## Supporting information

S1 File SPIRIT 2013 checklist. (DOC)

S2 File Study protocol (English translation). (DOCX)

S3 File Study protocol (Chinese version). (PDF)

## Abbreviations

APE: acquired premature ejaculation
CBT: cognitive behavioral therapy
CRC: Clinical research coordinators
CRF: case report form
FAS: full analysis set
FSDS-R-PE: female sexual distress scale-revised premature ejaculation version
IB: investigators brochure
IELT: intravaginal ejacula-tion latency time
LPE: lifelong premature ejaculation
NPE: natural variable premature ejaculation
PA: performance anxiety
PE: premature ejaculation
PEDT: premature ejaculation diagnostic tool
PP: per-protocol; SA: social anxiety
SPA: sexual performance anxiety
SPE: subjective premature ejaculation
SPIRIT: standard protocol items: recommendations for interventional trials
TMC: trial management committee
PDE5i: Phosphodiesterase Type 5 Inhibitor
ISSM: International Society for Sexual Medicine
