# Supplementary material for: Impact of cognitive behavioral therapy on premature ejaculation patients: A prospective, randomized controlled trial protocol
Source: PLoS One. 2023 Dec 27;18(12):e0295663. doi: 10.1371/journal.pone.0295663 (PMC10752513; doi:10.1371/journal.pone.0295663)
Supplement: S2 File — (DOCX) [file pone.0295663.s002.docx]

**Study title: Impact of Cognitive Behavioral Therapy (CBT) on Premature Ejaculation Patients: A Prospective, Randomized Controlled Trial Protocol**

**Background**

Premature ejaculation (PE) is a common male sexual dysfunction [1], with a global prevalence rate of approximately 20%-40% among adult males. PE negatively impacts the quality of patients' sexual life, affects their emotions, and may even lead to the development of psychological disorders, exacerbating the symptoms of premature ejaculation and creating a vicious cycle. The definition of premature ejaculation has been a subject of controversy [2]. In 2014, the International Society for Sexual Medicine (ISSM) defined premature ejaculation based on evidence-based research [3]: ejaculation always or nearly always occurs prior to or within approximately 1 minute of vaginal penetration (primary premature ejaculation), or clinically, the average intravaginal ejaculatory latency time (IELT) is significantly and distressingly reduced to about or less than 3 minutes (secondary premature ejaculation); in all or nearly all instances of vaginal penetration, ejaculation cannot be delayed or controlled, resulting in negative consequences such as distress, frustration, a sense of inadequacy, and avoidance of sexual contact. Overall, premature ejaculation encompasses three aspects: ejaculation occurring too early, poor ejaculatory control, and the presence of negative consequences.

Waldinger et al. [4] classified premature ejaculation (PE) based on etiology into two primary categories: lifelong PE (LPE) with a prevalence of 2.3% and acquired PE (APE) with a prevalence of 3.9%. In addition, there are two special types: natural variable PE (NPE) with a prevalence of 8.5%, and subjective PE/premature-like ejaculatory dysfunction (SPE) with a prevalence of 5.1%. In China, individuals with a complaint of PE account for approximately 26.0% of the population, with the prevalence of LPE being 12.3%, APE 18.8%, NPE 41.1%, and SPE 24.8% [5].

Urologists or specialists in sexual medicine are usually the first healthcare professionals consulted by patients with premature ejaculation. The diagnosis and evaluation primarily involve history taking, physical examination, ancillary investigations, scale assessments, and intra-vaginal ejaculation latency time (IELT) assessment. Among these, scales or questionnaires are the most widely used tools in clinical practice, providing reliable, interpretable, and standardized means of assessing and differentiating premature ejaculation. The Premature Ejaculation Diagnostic Tool (PEDT) is the most commonly used scale. Ancillary investigations mainly focus on determining the presence of comorbid conditions such as chronic prostatitis, erectile dysfunction, or endocrine disorders.

The etiology of premature ejaculation has not been definitively determined, leading to a variety of treatment approaches. Recent literature on premature ejaculation treatment can be classified into the following categories: psychological therapy, behavioral therapy, surgical treatment, local anesthesia medication, oral medication, and local treatments. Psychological therapy includes personalized interventions such as sexual health/technique knowledge education, psychological support, and cognitive improvement. Behavioral therapy typically involves gradually increasing levels of genital stimulation to help men better control ejaculation behavior. Local anesthesia can effectively increase the threshold of penile sensation, reduce penile sensitivity, improve IELT (intra-vaginal ejaculation latency time), and alleviate premature ejaculation symptoms. Oral medications include selective serotonin reuptake inhibitors (SSRIs) such as dapoxetine, tricyclic antidepressants (TCAs), phosphodiesterase-5 inhibitors (PDE-5i), alpha-blockers, and traditional Chinese medicine, which can improve symptoms. Local treatments involve external application of Chinese herbal medicine, electrophysiological therapy, and other methods. Surgical treatment is not recommended by the European Association of Urology, but clinical options may include procedures such as circumcision and selective dorsal neurectomy.

In recent years, several clinical studies in China have explored disease remote management using WeChat technology [6,7,8,9,10], including cardiovascular diseases, endocrine disorders, and postoperative urological rehabilitation. The results of these studies have shown that a patient-centered remote management model, based on in-hospital treatment and strengthened outpatient management, has improved treatment effectiveness and clinical outcomes to some extent compared to traditional conventional treatment models. Premature ejaculation is also a disease that relies on in-hospital treatment with auxiliary outpatient management. However, research exploring remote management for premature ejaculation in China is still lacking. This study aims to observe the clinical impact of remote management on premature ejaculation patients undergoing conventional treatment, explore suitable means and application scenarios for remote management, and provide references for the development of guidelines, consensus, and standards for remote management of premature ejaculation.

**Objectives and Outcomes**

Aim 1: To determine the efficacy of mobile-based CBT intervention compared to conventional treatment for patients with premature ejaculation (PE).
- Hypothesis 1a: a significant difference in the mean scores of the premature ejaculation diagnostic tool (PEDT) is expected between the two groups within 8 weeks.

Aim 2: To determine whether the duration of mobile-based CBT intervention has an impact on the efficacy for patients with PE.
- Hypothesis 2a: some significant differences in the PEDT scores, intravaginal ejaculatory latency time (IELT), and the Female Sexual Distress Scale-Revised premature ejaculation version (FSDS-R-PE) scores are expected between the two groups at 4 and 8 weeks.

**Outcomes**

**Primary outcome measures**

The mean PEDT scores of two groups of patients were recorded within 8 weeks. To assess treatment effectiveness, patients were evaluated using a medical questionnaire during each outpatient/telephone follow-up on day 1, at the end of week 4 and week 8 of the study. The questionnaire consisted of five questions with five different response categories (A=0, B=1, C=2, D=3, E=4) to evaluate the conditions over the past 6 months. Scores <8 indicated no problem; scores between 9 and 10 suggested a need for diagnosis at a hospital, while scores >11 were considered indicative of premature ejaculation. The PEDT scale has been widely used in China and has demonstrated good internal consistency, reliability, and validity. Studies have shown that the scale has good predictive ability in Chinese premature ejaculation patients.

**Secondary outcome measures**

(1) PEDT scores of the two groups at 4 and 8 weeks;

(2) IELT duration of the two groups at 4 and 8 weeks;

(3) FSDS-R-PE scores of the two groups at 4 and 8 weeks.

The FSDS-R-PE is a 13-item questionnaire with 5 response categories for each item (0=never, 1=rarely, 2=occasionally, 3=frequently, 4=always), designed to evaluate the sexual function status of female partners of men with premature ejaculation. A higher score indicates a higher level of dissatisfaction with sexual life.

**Trial design**

This study is a multicenter, randomized, controlled, open-label parallel-group exploratory trial, consisting of an intervention group and a standard treatment group. Participants will be randomly assigned in a 2:1 ratio to evaluate the efficacy of CBT in improving ejaculatory control in patients with PE. The study will be conducted from May 2023 to Dec 2024 at ten hospitals in China, primarily including the First Affiliated Hospital of Sun Yat-Sen University. The study protocol follows the Standard Protocol Items: Recommendations for Interventional Trials (SPIRIT).

**Eligibility criteria**

Patients meeting the following inclusion criteria were recruited: (1) male aged between 18-40 years; (2) in a stable heterosexual relationship with the same partner for at least 6 months; (3) diagnosed with PE according to the ISSM definition and not medically managed for at least 7 days; (4) able to use mobile devices such as smartphones and tablets; and (5) having read and agreed to sign the informed consent form.

Patients' exclusion criteria are as follows: (1) primary premature ejaculation patients; (2) patients taking medications such as amphetamines, dopamine, etc. that may cause PE; (3) patients with cognitive impairment, communication disorders, visual impairment, hearing impairment; (4) patients with other complex male genital diseases; (5) patients with malignant tumors; (6) patients participating in other clinical trials; (7) patients deemed unsuitable for this study by the researchers. This trial does not involve exploration of drug/medical device interventions.

Participants will be considered for withdrawal from the study under the following circumstances. However, even if participants do not withdraw their consent to participate in the study, they will no longer be considered as trial subjects and will not be included in the analysis of the study as soon as they withdraw from treatment under the following circumstances:

(1) If participants voluntarily withdraw (including dropping out, actively withdrawing, etc.);

(2) If the researcher requires the participant to withdraw due to disease deterioration or other reasons;

(3) If the researcher considers the participant no longer suitable to continue participating in the study.

The researchers will conduct preliminary eligibility screening and collect written informed consent from selected participants prior to baseline evaluation. If data collection is not included in the initial informed consent process of the main clinical trial, each participant in the ancillary study must sign a consent form.

**Group therapy**

Designated personnel at the trial center will input basic information of eligible patients into the randomization system according to the randomization table. A random number will then be generated. Patients who meet the inclusion criteria will undergo random allocation after obtaining signed informed consent.

Both patient groups received conventional treatments, including chemotherapy, traditional Chinese medicine, and instrument therapy, with treatment selection and plans based on the guidelines for diagnosis and treatment of premature ejaculation. To maximize the effectiveness of CBT interventions based on mobile devices in PE patients, we used remote management through social media software (WeChat) to address sensitive issues that may be difficult to reach in the population. We used the WeChat-MiniApp: "PE-CBT" developed by Hangzhou Kang Ming Information Technology Co., Ltd. to remotely manage the intervention group of patients. WeChat management mainly includes: (1) patients can achieve out-of-hospital coordination and management through WeChat and learn courses on cognitive-behavioral therapy; (2) researchers can track patient management through outpatient clinics (Fig 1). The specific course schedule is shown in the Fig 2. The entire course is divided into three stages. The stage 1 focuses on improving cognition, the stage 2 focuses on improving behavior, and the stage 3 focuses on reviewing.

**Patient compliance and withdrawal**

To ensure patients' compliance with the study, the research team will adopt a grid-based approach, such as telephone and internet reminders, to prompt patients to attend follow-up appointments at the outpatient clinic before the scheduled time. Meanwhile, during each follow-up visit, the research team will emphasize that patients in the intervention group should complete the relevant WeChat learning according to the requirements.

**Availability of investigational drugs/treatments**

This study does not involve the exploration of pharmaceutical/medical device interventions.

**Administration method and dose adjustment**

This study does not involve the exploration of pharmaceutical/medical device interventions.

**Adverse Event Reporting**

Any adverse event, whether causally related to the intervention or not, that occurred from the time the subject signed the informed consent and was enrolled in the trial until the end of the trial. Any medical occurrence prior to the administration of the intervention on day one medical condition or clinically significant laboratory abnormality that occurred prior to the administration of the intervention on Day 1 is considered pre-existing and must be documented in the case report The case report form must be documented. All AEs occurring after the administration of the intervention until the last day of the study (including follow-up, pause in the study) must be documented on the case report form. All AEs occurring after the administration of the intervention until the last day of the study (including follow-up, study pause) must be documented accordingly.

When completing the adverse event form, the investigator will use a scale of "1 to 5" to describe the severity of the adverse event.

To standardize the criteria, the intensity of the adverse event will be determined by reference to the CTCAE v3.0 grading definitions: Grade 1 (mild, asymptomatic or with mild signs; clinical or diagnostic observations only; no intervention required), Grade 2 (moderate, requiring minimal, local or non-invasive treatment; age-appropriate instrumental limitation of activities of daily living), Grade 3 (severe or clinically significant but not immediately life-threatening; hospitalization or extended hospitalization; disabling; limited ability to perform activities of independent living), Grade 4 (life-threatening, requiring urgent treatment), and Grade 5 (death).

**Data processing and storage**

Data collection will occur at four time points: baseline, day 1 of intervention period, 28±2 days, and 56±2 days. The collected data will be stored in the form of electronic case report forms (CRFs). Clinical research coordinators (CRC) will complete the electronic CRFs. Researchers will be informed of any changes/edit checks completed by the CRC and the system automatically. Throughout the study process, authorized researchers will be allowed to access the study computer and assess the data.

**Sample size**

This study adopted two control groups, namely, the remote management + conventional treatment group and the conventional treatment group, with a 2:1 ratio for enrollment. The primary research indicator was the PEDT score, with α=0.05 and β=0.2. Previous literature has shown that the PEDT score decreases by at least 2-3 points with psychological/behavioral therapy [20] and by at least 6-7 points with drug therapy [21, 22]. Given a maximum follow-up loss rate of 20% for each group, a target sample size of 300 patients, with 150 patients in each group, was determined.

**Statistical methods**

All data will be entered by two members separately and analyzed after confirming that the data is completely consistent. We will use SPSS 22.0 for statistical analysis. For metric data, Shapiro-Wilk test will be used to check for normality. If it meets the normal distribution, it will be described by mean ± standard deviation; otherwise, it will be described by median (P25, P75). Pearson correlation analysis will be used to evaluate the correlation, and P<0.05 will be considered statistically significant. Metric data of different groups will be described statistically using mean, standard deviation, median, minimum value, and maximum value. Count data of different groups will be described statistically using frequency (composition ratio). We will use exact probability calculation or non-parametric test to analyze the changes before and after intervention in each group. Regarding drop-out analysis, we will describe the actual number of enrolled subjects, excluded subjects, and drop-out subjects one by one, and analyze the specific reasons for drop-out and exclusion. The balance analysis of baseline values will be compared using analysis of variance or exact probability calculation for demographic data and other baseline indicators to measure the balance of each group. We will list adverse events that occurred during the trial and describe the changes in laboratory test results before and after the trial, and the relationship with the intervention when abnormal changes occurred.

**References**

[1] Russo A, Capogrosso P, Ventimiglia E, et al. Efficacy and safety of dapoxetine in treatment of premature ejaculation: An evidence-based review[J]. International Journal of Clinical Practice, 2016, 70(9):723-733.

[2] Hatzimouratidis, Konstantinos et al. “Erectile Dysfunction, Premature Ejaculation, Penile Curvature and Priapism EAU Guidelines on.” (2016).

[3] Serefoglu EC, Mcmahon CG, Waldinger MD. et al. An evidence-based unified definition of lifelong and acquired premature ejaculation: Report of the second international society for sexual medicine ad hoc committee for the definition of premature ejaculation[J]. Sexual Medicine, 2014, 2(2): 1423-1441.

[4] Waldinger, M.D., et al. The use of old and recent DSM definitions of premature ejaculation in observational studies: a contribution to the present debate for a new classification of PE in the DSM-V. J Sex Med, 2008. 5: 1079.

[5] Gao, Jingjing et al. “Prevalence and Associated Factors of Premature Ejaculation in the Anhui Male Population in China: Evidence-Based Unified Definition of Lifelong and Acquired Premature Ejaculation.” Sexual medicine vol. 5,1 (2017): e37-e43.

[6] Gang Zhao et al. Smartphone and social media-based cardiac rehabilitation and secondary prevention in China (SMART-CR/SP): a parallel-group, single-blind, randomised controlled trial[J]. Lancet Digital Health 2019; 1: e363–74

[7] Jinwen Wang et al. Efficacy of a WeChat based intervention to adherence to secondary prevention in patients undergoing coronary artery bypass graft in China: A randomized controlled trial[J]. Journal of Telemedicine and Telecare 2022, 28(9):653-661.

[8] Shen Junwen et al. The Efficacy of the WeChat App Combined with Pelvic Floor Muscle Exercise for the Urinary Incontinence after Radical Prostatectomy[J]. BioMed Research International Volume 2020, Article ID 6947839

[9] Meifang Xu et al. Effect of the WeChat Platform Health Management and Refined Continuous Nursing Model on Life Quality of Patients with Acute Myocardial Infarction after PCI[J]. Journal of Healthcare Engineering Volume 2021, Article ID 5034269

[10] Yanhui Liao et al. Effectiveness of the WeChat-based smoking cessation intervention (WeChat WeQuit program) in China: study protocol for a randomized controlled trial[J]. Addiction 116, 1279–1290
