# Supplementary material for: Impact of cognitive behavioral therapy on premature ejaculation patients: A prospective, randomized controlled trial protocol
Source: PLoS One. 2023 Dec 27;18(12):e0295663. doi: 10.1371/journal.pone.0295663 (PMC10752513; doi:10.1371/journal.pone.0295663)
Supplement: S3 File — (PDF) [file pone.0295663.s003.pdf]

附件 1-干预性研究方案

临床研究方案

研究标题：　　基于微信技术的远程管理对辅助改善早泄患者治疗效应的临床研究

版本号：　　V1.0

版本日期：　　2022 年 08 月 20 日

主要研究者：　邓春华 教授

所在科室：　　男科

2 研究背景及原理

早泄(premature ejaculation，PE)是是常见的男性性功能障碍疾病[1]，全球成年男性早泄的发病率约为 20%-40%。早泄影响患者性生活质量，还会影响患者情绪，甚至导致心理疾病的发生，加剧早泄的症状，形成恶性循环。早泄的定义一直都有争议[2]，2014 年国际性医学会(international society for sexual medicine，ISSM) 基于循证证据，将早泄定义为[3]：射精总是或者几乎总是发生在阴茎插入阴道之前或者插入阴道后约 1 分钟内（原发性早泄），或临床上平均阴道内射精潜伏时间(intravaginal ejacula- tion latency time，IELT) 显著而令人苦恼地减少，大约或者不足 3min（继发性早泄）；在所有或者几乎所有的阴道插入后射精无法延迟或控制；产生消极后果：如苦恼、烦恼、挫折感、避免性接触。总体包含 3 方面:射精过早、射精控制力差、产生消极后果。

Waldinger 等[4]将早泄进行分类,依据病因将其分为原发性早泄(lifelong PE，LPE，患病率 2.3%)和继发性早泄(acquired PE，APE，患病率 3.9%)，此外还有两种特殊类型：自然变异性早泄 (natural variable PE，NPE，患病率 8.5%) 以及主观性早泄/早泄样射精功能障碍(subjective PE/premature-like ejaculatory dysfunction，SPE，患病率 5.1%)。而在我国，主诉早泄的个体约占总体的 26.0%，其中 LPE12.3%、APE18.8%、VPE41.1%、SPE24.8%[5]。

早泄患者首诊医生通常选择泌尿男科（性医学科），其诊断与评估主要包括病史采集、体格检查、辅助检查、量表评估、IELT 评估等。其中量表是临床尚应用最广泛的调查问卷，为早泄的诊断与鉴别提供了可靠、可解释、标准化的评价手段，PEDT 使用最为广泛，而辅助检查主要是判断是否合并慢性前列腺炎、勃起功能障碍、内分泌疾病等。

早泄的病因尚未有明确定论，因此针对早泄的治疗方法也多种多样，查询近年来关于早泄治疗的文献报道，将其分为：心理疗法、行为疗法、手术治疗、局麻药物治疗、口服药物治疗、局部治疗等方面。心理疗法包括性健康/技巧知识教育、心理支持、认知改善等个性化心理干预；行为疗法一般利用各种逐渐增加对生殖器刺激程度的动作，使得男性能够更好地控制射精行为；局麻药可有效提高阴茎感觉阈值，降低阴茎敏感度，提高 IELT，改善早泄症状；口服药物包括达泊西汀等选择性 5-HT 受体抑制剂（SSRI），三环类抗抑郁药（TCA），磷酸二酯

酶5抑制剂（PDE-5i），α受体阻滞剂，中成药等改善症状；局部治疗包括中药外治、电生理理疗等方法；手术治疗未得到欧洲泌尿外科学会推荐，临床可以采用包皮环切术、阴茎背神经选择性切断术等。

近年来，国内开展了多项基于微信技术的疾病远程管理的临床研究[6,7,8,9,10]，包括心血管疾病、内分泌疾病、泌尿术后康复等，相关研究结果显示，以院内治疗为基础、以强化院外管理为目标的患者疾病远程管理模式，相较于传统的常规治疗模式，一定程度上提高了患者的治疗效应，改善了患者的临床结局。早泄也是一类以院内治疗为基础、院外管理为辅助的疾病，但国内早泄的远程管理探索研究尚属空白，期望通过本研究能够观察到远程管理对基于常规治疗的早泄患者的临床影响，且探索适宜远程管理的手段与应用场景，也期望为早泄的远程管理指南/共识/规范的制定提供参考。

3 研究目的及研究终点

3.1 研究目的

评价基于微信的远程管理对接受常规治疗的早泄患者临床治疗的影响

3.2 研究终点

主要研究终点及定义

比较两组间8周内早泄诊断量表（PEDT）平均得分的差异

次要研究终点及定义

比较两组间4周/8周时PEDT得分的差异；

比较两组间4周/8周时射精潜伏期（IELT）时长的差异；

比较两组间4周/8周时性苦恼量表早泄版（FSDS-R-PE）得分的差异

4 试验设计

4.1 总体试验设计

随机、平行、多中心研究设计

研究共计划入组300例，分为两组，1:1分配

探索性研究

4.2 试验时间

研究拟定随访8周

研究完成周期预计12个月内

病例资料预期采集时间范围从2022年9月30日至2023年10月9日，本院预计32例

4.3 试验简要流程图

阶段	筛选期	随访期		
访视	1	2	3	4
天	-7~~-1 天	1 天	28±2 天	56±2 天
知情同意	√			
入排	√			
人口学/疾病史/家族史	√			
体格检查/血尿常规/肝肾生化	√			
微信管理*		√	√	√
常规治疗**		√	√	√
量表评价***		√	√	√

*仅 A 组接受微信管理

**A/B 两组均接受常规治疗

***每次门诊/电话随访时完成 PEDT、FSDS-R-PE 量表评价

5 研究人群

5.1 入选标准

1.年龄在 18-40 周岁；

2.与同一异性伴侣保持稳定性关系至少 6 个月的男性；

3.根据 ISSM 定义诊断为 PE，且 7 天及以上未接受过医学管理；

4.能够使用手机、平板等移动终端设备；

5.已阅读受试者须知，并同意签署书面知情同意书。

5.2 排除标准

1. 原发性早泄患者；
2. 正在服用可能导致 PE 的药物如安非他明，多巴胺等的患者；
3. 存在认知障碍、交流障碍、视觉障碍、听力障碍的患者；
4. 合并其他复杂男科疾病的患者；
5. 恶性肿瘤患者；
6. 正在参与其他临床研究的患者；
7. 研究者认为不适合参与本研究的患者。

5.3 退出标准

1.患者自主退出（包括脱落、主动退出等）；

2.因疾病恶化等原因，研究者要求退出

3.研究者认为不适合继续参与本研究的患者。

6 研究治疗分组

A 组： 微信管理[1]+常规治疗[2]组

B 组： 常规治疗组

6.1 随机化分组

6.1.1 产生随机序列分配的方法

具体的随机分配方法通过建立随机系统表实施。

6.1.2 随机分配的隐藏

试验中心指定人员根据随机系统表，录入受试患者基本信息，产生随机号。凡符合纳入标准的患者，在获得签名的书面知情同意书后进行随机分配。

6.2 盲法及揭盲

非盲设计

7 研究程序

7.1 研究治疗期

7.1.1 A 组：微信管理+常规治疗：

从入组的第 1 天开始至第 8 周末，患者需接受：

1.微信管理

1.1 患者通过微信实现患者院外统筹管理,学习以认知行为疗法为方法学的系列课程：

1.1.1 PE 医学认知教育（标准化疾病教育）

1.1.2 PE 行为辅助训练（标准化行为训练）

1.1.3 PE 心理疏导（标准化心理辅导）

1.2 研究者通过门诊实现对患者管理的追踪：

1.2.1 PE 远程管理计划（告知患者如何接受微信管理）

1.2.3 PE 远程管理反馈（记录患者对管理的体会）

2.常规治疗：

包括化药、中药、器械治疗，治疗选择和治疗方案参照早泄诊疗指南

3.医学量表评价：

在研究的第 1 天、第 4 周末、第 8 周末，患者应接受医学量表评价，包括 PEDT、FSDS-R-PE 量表

7.1.2 B 组：常规治疗：

从入组的第 1 天开始至第 8 周末，患者需接受：

1.常规治疗：

包括化药、中药、器械治疗，治疗选择和治疗方案参照早泄诊疗指南

2.医学量表评价：

在研究的第 1 天、第 4 周末、第 8 周末，患者应接受医学量表评价，包括 PEDT、FSDS-R-PE 量表

7.2 研究性药物/治疗的供应

本研究不涉及探索药物/医疗器械干预

7.3 给药方法及剂量调整

本研究不涉及探索药物/医疗器械干预

7.4 伴随治疗、随访访视

试验期间无伴随治疗、无禁止行为

研究者应按研究方案要求执行患者的定期随访

7.5 患者依从性及退出

为保障患者研究依从性，研究团队将通过电话、网络等网格化方式，在随访时间前，及时提醒患者按期门诊随访。同时每次随访时，对 A 组患者强调应遵循要求完成微信相关学习。

7.6 方案偏离

出现下述情况时应及时报备方案偏离：

1.年龄超窗

2.随访超窗

8 评价

8.1 疗效评估

PEDT 量表为疗效评估的主要手段

8.2 安全性评估

8.2.1 基线体征和症状

本研究不涉及探索药物/医疗器械干预

8.2.2 实验室安全性评估

本研究不涉及探索药物/医疗器械干预

8.2.3 体格检查和生命体征

本研究不涉及探索药物/医疗器械干预

9 不良事件报告

9.1 不良事件

自受试者签署知情同意书并入选试验开始至试验结束，发生的任何不利的事

件，无论与干预手段是否有因果关系。在第一天给予干预手段以前发生的任何医疗情况或临床上明显的实验室异常，都认为是以前即已存在的，并须在病例报告表中记载。给予干预手段后直至研究的最后一天（包括追踪、研究的暂停期）发生的所有 AE 都必须做相应记录。

9.2 不良反应严重程度判定标准：

在填写不良事件表时，研究者将使用“1～5 级”来描述不良事件的严重程度。为统一标准，不良事件强度参考 CTCAE v3.0 的分级定义进行判断：1 级（轻度，无症状或有轻度体征；仅有临床或诊断观察结果；无需介入治疗）、2 级（中度，需要最低程度、局部或非侵袭性的治疗，与年龄相适应的工具性日常生活活动能力受限）、3 级（严重或临床显著意义但非即刻危及生命；住院或延长住院时间；致残；自理生活活动能力受限）、4 级（危及生命，需要紧急治疗）、5 级（死亡）。

9.3 不良事件与干预手段关系的判断标准：

研究者应对不良事件和干预手段以及合并用药之间可能存在的关联作出评估，参照以下 5 级分类“肯定有关、可能有关、可能无关、无关、无法判定”标准评定。肯定有关（用药及反应发生时间顺序合理。停药后反应停止或迅速减轻或好转。再次使用，反应再现。同时有文献资料佐证。并已除原患疾病等其他混杂因素的影响）、可能有关（用药与反应发生时间关系密切，同时有文献资料佐证。但引发不良反应的药品不止一种，或原患疾病病情进展因素不能除外）、可能无关（不良反应与用药时间相关性不密切，反应表现与该药的已知不良反应不相吻合，原患疾病发展同样可能有类似临床表现）、无关（这一类型的不良事件，在评审时经过细致的医学考虑后，被明确地、不可争议地认为是由外因疾病、环境等引起以及不符合有关、可能有关和可能无关类别下的衡量标准。）、无法评定（缺项太多，因果关系难以定论，资料又无法补充）。

9.4 严重不良事件的定义：

不良事件符合下面 1 条或 1 条以上标准时归为 SAE。①死亡、②致癌、致畸、致出生缺陷、③对生命有危险或能够导致人体永久的或显著的伤残、④对器官功能产生永久的损伤、⑤导致住院治疗或住院时间延长。

9.5 严重不良事件的记录与报告：

出现严重不良事件时应在 24 小时内向伦理委员会和主要研究者报告，并同

时上报给 SFDA 安监司和发生地的省级食品药品监督管理局，并填写严重不良事件表，记录 SAE 的发生时间、严重程度、持续时间、采取的措施和转归。

10 数据处理及保存

10.1 数据的记录：受试者相关所有数据的记录必须及时、准确、完整、规范、真实。对任何数据更正时只能划线，旁注改后的数据，说明理由，由研究者签名并注明日期，不得擦涂、覆盖原始记录。

10.2 数据的监查：指定人员完成审核研究记录，确认数据记录准确、规范、完整、真实。

10.3 数据的检查和录入：研究者应对研究源数据进行检查、确认后，录入数据库供统计分析。

10.4 资料存档：结束后应将研究资料存档。

11 质量管理

11.1 研究者必须保证数据真实、完整、准确。

11.2 研究记录所有项目均需填写，不得空项、漏项(无记录的空格按要求填写)做任何更正时只能划线，旁注改后的数据，说明理由，由研究者签名并注明日期，不得擦涂、覆盖原始记录。

11.3 研究者应实验室检查项目齐全

11.4 研究者在试验过程中到试验单位检查受试者的知情同意及筛选纳入情况。

11.5 研究者确认所有病例报告表填写正确并与原始资料一致。

11.6 研究者应注意所有错误或遗漏均以改正或注明，经研究者签名并注明日期。

11.7 研究者确认所有不良事件均以记录在案，严重不良事件已做出报告并记录在案。

12 统计分析

12.1 样本量确定

本研究采用两组对照，远程管理+常规治疗组与常规治疗组按照 2:1 比例入组，主要研究指标为 PEDT 得分，其中 α=0.05，β=0.2，既往文献发现使用药物治疗后 PEDT 得分降低至少 2.3，使用心理治疗后 PEDT 得分降低至少 6.7，假设研究结束后两组患者平均 PEDT 得分相差 4.5，即 δ=4.5，s 取 11.65，得出常规治疗组最小样本量为 80，远程患者管理+常规治疗组最小样本量为 160 例，考虑

失访率 20%，两组合计应至少纳入样本 300 例。

12.2 分析方法

所有数据均由 2 名成员分别录入，确认数据完全一致后进行分析。采用 SPSS22.0 进行统计分析。计量资料均采用 Shapiro-Wilk 法进行正态性检验，符合正态分布的数据，采用均数±标准差进行统计描述；不符合正态分布的数据采用中位数（P25，P75）进行统计描述，通过 Pearson 相关性分析进行相关度分析，以 P＜0.05 为差异有统计学意义。不同组别的计量资料将采用均数、标准差、中位数、最小值、最大值进行统计描述。不同组别的计数资料采用频数（构成比）进行统计描述。各组干预前后的变化采用确切概率计算或非参数检验。脱落分析：实际入组受试者例数，剔除例数，脱落例数逐一进行统计描述，分析具体脱落、剔除原因。基础值的均衡性分析：采用方差分析或确切概率计算比较人口学资料和其它基础值指标，以衡量各组均衡性如何。列表描述本次试验所发生的不良事件；实验室检查结果在试验前后正常/异常的变化情况以及发生异常改变时与干预手段的关系。

13 中期分析

无。

14 数据监查委员会

无。

15 道德伦理

本临床试验遵循赫尔辛基宣言和中国有关临床试验研究规范、法规进行。在试验开始之前，临床试验方案及其修订申请、病例报告表、知情同意书等均应取得中山大学附属第一医院伦理委员会的书面批准后，方可执行。每一例受试者入选本试验前，研究者均应向其完整、全面地介绍研究的目的、程序及参加试验的收益和可能的风险等。研究者在试验前获得双方共同签名并注明日期的知情同意书。知情同意书原件由研究者保存，签名页副本由受试者保存。受试者可以随时退出本次临床研究。

16 研究结束的定义

完成第 8 周末末次随访即研究结束

17 试验组织架构

项目成员	编号	姓名	科室	职称	手机	项目分工
	1	邓春华	男科	主任医师	13501519349	PI
	2	孙祥宙	男科	主任医师	13600476823	Sub-I
	3	涂响安	男科	主任医师	13822196816	Sub-I
	4	高勇	男科	副主任医师	15899950861	Sub-I
	5	张亚东	男科	副主任医师	13560411398	Sub-I
	6	庄锦涛	男科	副主任医师	13632319746	Sub-I
	7	杨其运	男科	主治医师	13580385866	Sub-I
	8	韩大愚	男科	住院医师	13430377557	Sub-I
	9	万子	男科	主治医师	15918642886	Sub-I
	10	蔡鸿才	男科	住院医师	15920995848	Sub-I

18 保密及数据安全

参加试验及在试验中的患者资料均属保密。患者的医学数据将以研究编号数字而非患者的姓名加以标识。可以识别患者身份的信息将不会透露给研究小组以外的成员，除非获得患者的许可。所有的研究成员和研究申办方都被要求对患者的身份保密。患者的档案将保存在有锁的档案柜中，仅供研究人员查阅。为确保研究按照规定进行，必要时，政府管理部门或伦理审查委员会的成员按规定可以在研究单位查阅患者的个人资料。这项研究结果发表时，将不会披露患者个人的任何资料。

19 保险和赔偿

本研究无保险和赔偿

20 出版说明

暂无出版计划

21 参考文献 （请附上相关文献）

[1] Russo A, Capogrosso P, Ventimiglia E, et al. Efficacy and safety of dapoxetine in treatment of premature

ejaculation: An evidence-based review[J]. International Journal of Clinical Practice, 2016, 70(9):723-733.

[2] Hatzimouratidis, Konstantinos et al. “Erectile Dysfunction, Premature Ejaculation, Penile Curvature and Priapism EAU Guidelines on.” (2016).

[3] Serefoglu EC, Mcmahon CG, Waldinger MD. et al. An evidence-based unified definition of lifelong and acquired premature ejaculation: Report of the second international society for sexual medicine ad hoc committee for the definition of premature ejaculation[J]. Sexual Med- icine, 2014, 2(2) : 1423-1441.

[4] Waldinger, M.D., et al. The use of old and recent DSM definitions of premature ejaculation in observational studies: a contribution to the present debate for a new classification of PE in the DSM-V. J Sex Med, 2008. 5: 1079.

[5] Gao, Jingjing et al. “Prevalence and Associated Factors of Premature Ejaculation in the Anhui Male Population in China: Evidence-Based Unified Definition of Lifelong and Acquired Premature Ejaculation.” Sexual medicine vol. 5,1 (2017): e37-e43.

[6] Gang Zhao et al. Smartphone and social media-based cardiac rehabilitation and secondary prevention in China (SMART-CR/SP):a parallel-group, single-blind, randomised controlled trial[J]. Lancet Digital Health 2019; 1:e363–74

[7] Jinwen Wang et al. Efficacy of a WeChat based intervention to adherence to secondary prevention in patients undergoing coronary artery bypass graft in China: A randomized controlled trial[J]. Journal of Telemedicine and Telecare0(0)1–9

[8] Shen Junwen et al. The Efficacy of the WeChat App Combined with Pelvic Floor Muscle Exercise for the Urinary Incontinence after Radical Prostatectomy[J]. BioMed Research International Volume 2020, Article ID 6947839

[9] Meifang Xu et al. Effect of the WeChat Platform Health Management and Refined Continuous Nursing Model on Life Quality of Patients with Acute Myocardial Infarction after PCI[J]. Journal of Healthcare Engineering Volume 2021, Article ID 5034269

[10] Yanhui Liao et al. Effectiveness of the WeChat-based smoking cessation intervention (WeChat WeQuit program) in China:study protocol for a randomized controlled trial[J].Addiction 116, 1279–1290

22 附录

一、早泄诊断量表（PEDT）

请根据过去 6 个月内的情况评估	
1. 性交时想推迟射精有多大困难？	◎没有困难<br>◎有点困难<br>◎中等困难<br>◎非常困难<br>◎完全无法延迟
2.射精发生在想射精之前几率？	◎（几乎）没有<br>◎不经常<br>◎约五成<br>◎多数时候<br>◎几乎/总是
3.是否收到很小的性刺激就会射精？	◎（几乎）没有<br>◎不经常<br>◎约五成<br>◎多数时候<br>◎几乎/总是
4. 是否对过早射精感到沮丧？	◎完全没有<br>◎有点<br>◎一般<br>◎偶尔<br>◎经常
5. 射精时间造成伴侣不满意，你对此担心吗？	◎完全没有<br>◎有点<br>◎一般<br>◎偶尔<br>◎经常

二、FSDS-R-PE 量表：

您经常感到:	0=从不,1=很少,2=偶尔,3=经常,4=总是				
1. 对您的性生活感到苦恼	0	1	2	3	4
2. 对您的两性关系不满意	0	1	2	3	4
3. 对实施性生活困难而感到内疚	0	1	2	3	4
4. 对您的性生活问题感到沮丧	0	1	2	3	4
5. 对性生活感到压力	0	1	2	3	4
6. 因性问题而感到自卑	0	1	2	3	4
7. 对性生活表示担忧	0	1	2	3	4
8. 对性生活信心不足	0	1	2	3	4
9. 对当前的性取向后悔	0	1	2	3	4
10. 对性问题感到尴尬	0	1	2	3	4
11. 对您的性生活不满意	0	1	2	3	4
12. 对您的性生活感到愤怒	0	1	2	3	4
13. 被您伴侣的早泄所困扰	0	1	2	3	4
